# Optimal dietary patterns designed from local foods to achieve maternal nutritional goals

**DOI:** 10.1186/s12889-018-5369-x

**Published:** 2018-04-04

**Authors:** Jofrey Raymond, Neema Kassim, Jerman W. Rose, Morris Agaba

**Affiliations:** 1 School of Life Science and Bioengineering, Nelson Mandela African Institution of Science and Technology (NM-AIST), P.O. Box 447, Arusha, Tanzania; 2 SolBridge International School of Business, Woosong University, Daejeon, Republic of Korea

**Keywords:** Optimal dietary patterns, Local foods, Maternal undernutrition, Linear goal programming, Micronutrient malnutrition, Tanzania

## Abstract

**Background:**

Achieving nutritional requirements for pregnant and lactating mothers in rural households while maintaining the intake of local and culture-specific foods can be a difficult task. Deploying a linear goal programming approach can effectively generate optimal dietary patterns that incorporate local and culturally acceptable diets. The primary objective of this study was to determine whether a realistic and affordable diet that achieves nutritional goals for rural pregnant and lactating women can be formulated from locally available foods in Tanzania.

**Methods:**

A cross sectional study was conducted to assess dietary intakes of 150 pregnant and lactating women using a weighed dietary record (WDR), 24 h dietary recalls and a 7-days food record. A market survey was also carried out to estimate the cost per 100 g of edible portion of foods that are frequently consumed in the study population. Dietary survey and market data were then used to define linear programming (LP) model parameters for diet optimisation. All LP analyses were done using linear program solver to generate optimal dietary patterns.

**Results:**

Our findings showed that optimal dietary patterns designed from locally available foods would improve dietary adequacy for 15 and 19 selected nutrients in pregnant and lactating women, respectively, but inadequacies remained for iron, zinc, folate, pantothenic acid, and vitamin E, indicating that these are problem nutrients (nutrients that did not achieve 100% of their RNIs in optimised diets) in the study population.

**Conclusions:**

These findings suggest that optimal use of local foods can improve dietary adequacy for rural pregnant and lactating women aged 19–50 years. However, additional cost-effective interventions are needed to ensure adequate intakes for the identified problem nutrients.

## Background

Adequate nutrient intake during the life stages of pregnancy and lactation is important for the proper fetal growth and production of breast milk. Poor nutrition during pregnancy and lactation periods put mothers and their growing children at a greater risk of disease, mental disorders and death. Nutrient needs during these periods are relative higher compared to women who are not pregnant or lactating [1, 2]. Based on dietary reference intakes set by the World Health Organization (WHO), energy needs are 13% higher during pregnancy and 25% higher during lactation, and protein needs are 54% higher during both periods compared to non-pregnant and non-lactating woman [3–5]. Likewise, the requirements for several essential micronutrients such as folate and iron during pregnancy, and vitamin A, vitamin C, vitamin B6, iodine, calcium and zinc during lactation are ≥50% [6]. Poor dietary intakes during this critical period may increase the risk of undernutrition, morbidity and mortality of infants and women.

Maternal undernutrition commonly co-exist in pregnant and lactating women, especially in lower-income settings [6]. Evidence shows that between 5% and 20% of African women have a low body mass index (BMI) associated with chronic hunger. The prevalence of anaemia ranges from 21 to 80% across the continent, with equally high values for both vitamin A and zn deficiency levels [7]. In Tanzania, for instance, more than 5.5% of women aged 15–49 years are underweight (with 0.4% severely underweight) [8]. Furthermore, among women aged 15–49 years, 30% are suffering from iron deficiency, 36% from iodine deficiency, 37% from vitamin A deficiency, and 40% from anaemia [9].

Maternal malnutrition is responsible to a great extent for millions of childhood deaths and incidences of diseases every year in lower-income countries [10, 11]. Statistics show that more than 1 million infant deaths occurs within the first month, including mortality rate of 50% within one day of delivery in Sub-Saharan Africa (SSA). The highest rates of neonatal mortality in the world with a range of 1 in 16 to 1 in 45 from causes associated with childbirth are in SSA. Also, about 1 million stillbirths cases occur annually in the region [12]. In Tanzania, about 40,000 neonatal deaths with mortality rate of 20 are reported annually [13]. The survivors are likely to suffer permanent developmental impairment that can stifle cognitive performance, physical growth and school achievements, consequently limiting child’s ability to benefit from economic opportunities [14]. These troubling long-term effects underscore the need for a continued focus on the crucial 1000 days window during pregnancy and the first 2 years of child’s life.

In order to tackle the problem of undernutrition, several interventions are being practiced globally. Some of these interventions include maternal multiple micronutrient supplementation, calcium supplementation to mothers at risk of low intake, maternal balanced energy protein supplementation and universal salt iodization [6]. Addressing the issue of cultural behavior to promote dietary diversification is one of the long-term strategies for improving maternal nutrition. Consumption of locally available nutrient-dense foods is being emphasized as sustainable option for tackling the co-existence of undernutrition in vulnerable populations [6]. This alternative, however, requires resolving barriers that may be posed by factors like limited access and high price of such foods, especially in lower-income settings. Optimisation based on linear programming (LP) techniques can serve a purpose of resolving the identified barriers. The LP approach can take into account the multiple factors such as food costs and cultural factors in ensuring the development of affordable and culturally acceptable food patterns [15]. The objective of the present study was to determine whether a realistic and affordable diet formulation that achieves nutritional goals for rural pregnant and lactating women can be formulated from locally available foods in Tanzania.

## Methods

### Site description

The present study was carried out in six randomly selected villages in Bahi district, central zone of Tanzania. This district was purposively selected because it is located in a semi-arid area with a low ecosystem productivity. The district is a typical rural area experiencing rapid growth and change in population characteristics. The area is also susceptible to climate and weather variability that may affect traditions and local food production and consumption patterns which consequently impact the nutrition security.

### Survey design

A cross-sectional survey was carried out from September to December 2015 to assess dietary intake patterns of 150 pregnant and lactating mothers. The written informed consent was obtained from each participant. A research protocol of the present study was approved by the Research Committee at the School of Life Sciences and Bioengineering, The Nelson Mandela African Institution of Science and Technology (NM-AIST). A market survey was also conducted to determine the cost of edible portion of local foods that are frequently consumed in the study area. Data from dietary and market surveys were then used to drive LP model parameters for diet optimisation. All the analyses were done using Microsoft Excel 2013 and linear program solver version 1.9.4 to generate optimal nutrition solutions.

### Participants and sampling

**Fig. 1:**
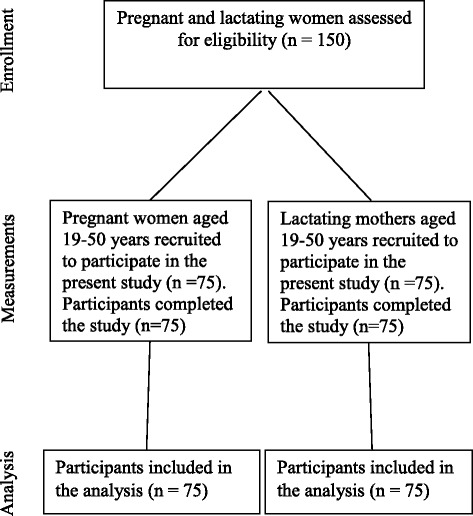
Participants’ flow diagram

Six villages were randomly selected from 59 villages in Bahi District and 25 mothers were randomly selected from each village. In the present study we enrolled a total of 75 pregnant women and 75 lactating mothers as described in the participants’ flow diagram (Fig. 1). The sample size was previously drawn from a list using ProPAN sampling guidelines at 95% confidence interval (CI) [16]. The intention was that the overall sample size would be split equally among the villages. The inclusion criteria were that a woman is pregnant or is having a child aged below 6 months and have agreed to participate in the study survey. In addition, the woman was not supposed to be suffering from any ailment that may affect her dietary intakes.

### Anthropometric measurement

Weights of the women were measured to the nearest 0.1 kg using a battery powered digital scale and heights were measured to the nearest centimeter using a height scale using standard anthropometric techniques [17]. For weight and height measurements, study subjects removed their shoes and jackets and wore light clothing. Body mass index (BMI) of each participant was calculated by dividing the weight in kilogram to the height in meter squared (Kg/m2). BMI less than 18.5 was considered as underweight (malnourished) based on WHO BMI classification [18]. We did not calculate the BMI for pregnant women.

### Dietary consumption pattern

Dietary data were collected using 24-h dietary recalls, weighed dietary record (WDR) and 7 days food records as described in previous studies [19, 20]. The WDR method was used to collect food consumption data, whereas, 24-h dietary recalls and 7-day food records were used to describe food patterns per week. No food portion size was collected using 24-h recall and 7-day food records.

A single 24-h recall was done to collect data on foods and beverages consumed within 24 h before the WDR day. During the interview, each participant was asked to recall all foods and beverages she consumed in the past 24 h. In some cases, pictures and food models were shown to a participant so that she could identify and point out food items consumed. Participants were also asked to mention other foods and beverages that were not included in our food models and pictures.

In the case of WDR method, all foods and beverages consumed by the women were weighed by using electronic kitchen scale (CAMRY, Model EK3131, precision ±2 g). A field researcher was present at a household for 12 h of the day to observe and weigh the amount of all foods and beverages served and consumed by the participating woman. Furthermore, participants were asked to estimate the amount of foods consumed after the observer has left the household. All composite foods and dishes were broken down into their constituent ingredients such as oil, sugar, salt, tomato, carrots, onions and so forth. The weighed dietary records were collected on all days of the week to represent the effect of any day of the week on dietary intakes of subjects.

In the case of 7-days food records, a self-administered 7-day food tally was done to collect data on the frequency of foods and beverages consumed after the WDR day. In this approach, participants were asked to record all foods and beverages consumed during the 7-days period. For illiterate participants (25%) and those who forgot (5%) to record the consumed foods, a 12-h recall was conducted for 7 days. About 70% of participants provided 7 days food records. Finally, dietary records obtained from women were classified by age group and physiological status [21] based on the categorization of recommended nutrient intakes (RNIs) for pregnant and lactating women [3–5].

### Market survey

A market survey was also conducted in one local market and small shops which were frequently used by participants in the village. Only one price per food item was collected in small shops available in each village. If the survey location is a market with multiple vendors, three different prices per food item were collected, and an attempt was made to include both the highest and lowest prices across all vendors. For cooked composite dishes, each raw ingredient was weighed and their costs were summed to obtain the cost for all ingredients. The cost of each food item was linked to the food composition database for our linear programming models.

### Preparation of model parameters

The preparation of LP model parameters was done in Microsoft Excel 2013. These parameters included food subgroups, a median serving size for each food subgroup, the lower, median, and upper limits of serving sizes. Food subgroups were defined by grams (serving sizes) of individual foods that were consumed by ≥5% of women and nutrient-dense foods consumed by ≤5% of participants. The serving size for each food subgroup was defined by the median serving size for women who consumed the food in each group. The lower, median and upper limits of serving sizes were defined as 10th, the 50th and 90th percentile of food patterns for each study group. These LP parameters were used to set up the LP models for the analyses in linear program solver. The diets were modelled for a 7-day period.

### Food composition database

Dietary intakes of nutrients were estimated based on Tanzania food composition tables [22] and United States Department of Agriculture food composition database [23]. In some cases, the amount of foods served and consumed were converted to their raw form using cooked to raw conversion factors so as to match nutrient values in food composition databases [16]. In the present study, a total of 35 food items appeared in the dietary records and were categorized into 12 food groups as described by FAO [24]. The nutrient profiles were established based on WDR using median proportion size per food item. Nutrient values for the foods consumed in cooked state were adjusted for cooking losses using USDA retention factors to avoid overestimation [25]. Nutrient profiles were calculated separately for each group and were used as input data for our LP models.

### Objective function of LP models

The objective function was to minimize the deviation from the population’s food consumption patterns for 12 food groups while simultaneously meeting the required dietary standards. Based on the study by Okubo and colleagues [21], the objective function is represented by:

$$ Y=\sum \limits_{i=1}^n\left|\left({Q}_i^{opt}-{Q}_i^{obs}\right)/{Q}_i^{obs}\right| $$

where Y denotes the objective function to minimize, and ${Q}_i^{opt}$denotes the quantity (g) of food subgroup i in the optimised food intake pattern, and ${Q}_i^{obs}$ denotes the median quantity (g) of food subgroup i in the observed food intake pattern. Since, the absolute value Y was nonlinear, we had to transform Y into a linear function using goal programming approach described in previous studies [21, 26, 27]. New decision variables ≥0, representing positive (${d}_1^{+}$ to${d}_{40}^{+}$) and negative (${d}_1^{-}$ to${d}_{40}^{-}$) deviation from the quantity of the observed food subgroup were created and defined as follows:

$$ {\displaystyle \begin{array}{l}\mathrm{If}\;{Q}_i^{opt}<{Q}_i^{obs}\;\mathrm{then}\kern0.24em {d}_i^{-}=\left({Q}_i^{obs}-{Q}_i^{opt}\right)/{Q}_i^{obs}\;\mathrm{and}\;{d}_i^{+}=0.\\ {}\mathrm{If}\;{Q}_i^{opt}>{Q}_i^{obs}\;\mathrm{then}\kern0.24em {d}_i^{+}=\left({Q}_i^{opt}-{Q}_i^{obs}\right)/{Q}_i^{obs}\  and\ {d}_i^{-}=0.\\ {}\mathrm{If}\;{Q}_i^{opt}={Q}_i^{obs}\;\mathrm{then}\kern0.24em {d}_i^{-}=0,\mathrm{and}\kern0.24em {d}_i^{+}=0.\\ {}\mathrm{Subject}\ \mathrm{to}:{d}_i^{+}-{d}_i^{-}=\left({Q}_i^{obs}-{Q}_i^{opt}\right)/{Q}_i^{obs}.\end{array}} $$

The new linear function termed Y′ was expressed as the sum of the deviational variables and minimized as follows:

$$ \mathit{\operatorname{Minimize}}\kern0.5em {Y}^{\hbox{'}}\kern0.5em =\sum \limits_{i=1}^n\kern0.5em \left({d}_i^{-}\kern0.5em {d}_i^{+}\right) $$

where ${d}_i^{-},{d}_i^{+}$ are negative and positive deviational variables representing deviations from the i^*th*^ goal. For the recommended nutrient intake (RNI) constraints positive deviation ${d}_i^{+}$ = 0, while for the (upper limit) UL constraints negative deviation ${d}_i^{-}$= 0. Each food subgroup in the objective function was linked to the nutrient profile and cost databases established for the present study. The model calculated the intake of food groups and subgroups at all times and checked whether nutritional constraints were satisfied. The observed intake of each food group or subgroup was defined according to the median intake of that food group or subgroup across the whole population.

### Nutritional constraints for LP models

We introduced a set of constraints to our LP models to minimise the gap between observed and optimised food intake patterns. The intention was to ensure that the solution selected by a model satisfies all of the specified constraints. Based on the previous studies [21, 27], upper limits were set on the quantity of each food to ensure that program models did not exceed the usual intake of foods in the study population. These limits were derived from the actual intake patterns for each age group as reported in a 12-h weighed dietary record (WDR). The dietary intake of all foods from each food subgroup was required not to exceed the 90th percentile of intake for each group. Food intakes were constrained within the range from the 10th percentile to 90th percentile of the observed intakes for each group. In this study, however, the intake of fish, meat, vegetables, fruits and seeds was very low. We, therefore, had to exceptionally set a maximum intake level to ≥90th percentile for each group aiming to promote consumption of these nutrient-dense foods.

Nutritional constraints were included to ensure that the nutritional content of each optimised food intake pattern was equal to or greater than the required value based on the RNIs for pregnant and lactating women. The recommended nutrient intakes (RNIs) for all the 23 studied nutrients published by WHO [3–5] was the criterion for determining whether each nutritional goal had been attained by the optimised food intake pattern. In addition, food cost constraint was set to ensure that the cost of the optimised food intake pattern was equal to the maximum cost of all diets consumed by women in each group.

## Results

### Characteristics of participants

The total of one hundred and fifty women (pregnant and lactating women) from predominantly rural households in Bahi district located in central zone of Tanzania were surveyed. Among the 150 subjects, 50% were pregnant mothers aged 19–50 years and the other 50% were lactating mothers with infants aged below six months. Thirty percent of all lactating mothers were underweight (adult BMI < 18.5).

### Observed food patterns

Generally, a total of 35 food items were reported in the dietary data that were collected from pregnant and lactating women in the study area. The observed diets that were frequently consumed in the study area were mainly cereal based. Animal-source foods were less consumed by all studied mothers. Out of 35 food items, 12 major food groups were formed based on FAO guidelines for measuring household and individual dietary diversity [24]. Major food groups that were formed include; cereal and cereal products; roots, tubers and plantations; legumes, nuts and seeds; vegetables; fruits; meat, poultry and insects; eggs; fish and seafood; milk and dairy products; fat and oils; sweets; spices, condiments and beverages. Furthermore, the 10th, 50th and 95th percentile of intake of all candidate foods that could be successfully promoted were established for both pregnant and lactating women in the study area (Table 1).

**Table 1:** Food groups and subgroups (g/day) with lower or upper limit constraints included in linear programming optimisation models

		Pregnant women (*n* = 75)			Lactating mothers (*n* = 75)		
		Lower	Average	Upper	Lower	Average	Upper
Food group	Food subgroup	P10	P50	P90	P10	P50	P90
Cereals and cereal products		847.6	1375.6	2028.1	633.2	1201.3	1636.1
	Refined grains	262.3	398.2	613.7	327.1	566.0	729.4
	Whole grains	585.3	977.5	1414.4	306.1	635.3	906.7
Roots, tubers and plantains		133.1	230.8	418.6	242.0	406.5	611.5
	Banana plantain	53.1	70.8	88.5	106.0	212.0	296.8
	Irish potatoes	80.0	160.0	330.2	49.3	50.0	71.0
	Cassava	0.0	0.0	0.0	74.4	124.0	223.2
	Sweet potatoes	0.0	0.0	0.0	12.3	20.5	20.5
Pulses, nuts and seeds		37.4	54.5	90.8	49.9	64.8	114.7
	Bambara nuts	12.0	16.0	28.8	26.4	32.0	54.4
	Dried beans	22.2	30.3	50.0	20.5	25.0	50.0
	Groundnuts	3.2	8.2	12.0	3.0	7.8	10.3
Vegetables		153.7	187.9	408.0	309.0	413.8	633.4
	Green and yellow vegetables	152.1	182.6	392.0	307.4	409.0	622.7
	Other vegetables	1.6	5.3	16.0	1.6	4.8	10.7
Fruits		4.2	8.0	10.0	2.2	4.0	8.0
	Baobab powder	4.2	8.0	10.0	2.2	4.0	8.0
	Lemon juice	0.0	0.0	0.0	0.0	0.0	0.0
	Ripe banana	0.0	0.0	0.0	0.0	0.0	0.0
Meat, poultry and insects		7.4	20.0	20.1	7.2	20.0	25.0
	Beef	7.4	20.0	20.1	7.2	20.0	25.0
Eggs	Egg	0.0	0.0	0.0	0.0	0.0	0.0
Fish and seafood		25.0	42.2	62.3	20.0	29.0	49.5
	Fish, dried	0.0	0.0	0.0	0.0	0.0	0.0
	Sardines, dried	25.0	42.2	62.3	20.0	29.0	49.5
Milk and dairy products		15.6	30.3	45.0	0.0	0.0	0.0
	Cow milk	15.6	30.3	45.0	0.0	0.0	0.0
Fats and oils		2.2	7.0	19.0	2.7	7.6	19.0
	Vegetable oil	2.2	7.0	19.0	2.7	7.6	19.0
Sweets		14.2	23.3	50.0	14.0	21.0	28.0
	Sugar	14.2	23.3	50.0	14.0	21.0	28.0
Spices, condiments and beverages		1.1	3.2	8.4	1.1	2.5	5.1
	Table salt	0.9	2.3	7.0	0.7	1.9	4.3
	Tea, black	0.2	0.9	1.4	0.4	0.6	0.8

### Optimal food intake patterns

Optimal dietary intake patterns for pregnant women aged 19–50 years and lactating mothers aged 19–50 years were generated and compared to their observed food intake patterns (Table 2). Our analysis showed that the optimal intakes of cereal and or cereal products were significantly lowered. The modification was from 1255.3 g to 434.3 g for pregnant women, and from 1201.3 g to 306.1 g for lactating mothers. This means that 821 g and 895.1 g were reduced from observed intakes of cereals and or cereal products for pregnant women and lactating mothers, respectively. On the other hand, we observed that the optimal intakes of other ingredients like vegetables, fruits, seeds and animal source foods increased significantly. For example, the difference increase between observed and optimal vegetables intake patterns was 220.1 g for pregnant women and 219.6 g for lactating mothers. Furthermore, the difference between observed and optimised intake patterns of fruits was 8 g for pregnant women and 4 g for lactating mothers. Likewise, the difference of 0.1 g and 5 g for pregnant women and lactating women, respectively was observed in meat intakes. In addition, the difference of 14.2 g and 27.5 g for pregnant women and lactating mothers, respectively was observed in pulses, nuts and seeds intakes. Also, the difference between observed and optimal fish and seafood intake patterns were 20.1 g for pregnant women and 79.5 g for lactating mothers. Moreover, the difference between observed and optimal intakes of milk and dairy products was 14.7 g for pregnant women and zero intake for lactating mothers. Similarly, the difference of 2.1 g and 1.8 g for pregnant and lactating women, respectively was observed in spices, condiments and beverages intakes. Additionally, the difference between observed and optimal fats/oils intake pattern was 4.8 g for pregnant women and 4.9 g for lactating mothers.

**Table 2:** Comparison of food quantities (g/day) between observed and optimised food intakes

		Pregnant mothers 19–50 years (*n* = 75)		Lactating mothers 19–50 years (*n* = 75)	
Food group	Food subgroup	Observed	Optimised	Observed	Optimised
Cereals and cereal products		1255.3	434.3	1201.3	306.1
	Refined grains	277.9	0.0	566.0	0.0
	Whole grains	977.5	434.3	635.3	306.1
Roots, tubers and plantains		230.8	80.0	406.5	599.0
	Banana plantain	70.8	0.0	212.0	296.8
	Irish potatoes	160.0	80.0	50.0	71.0
	Cassava tubers	0.0	0.0	124	223.2
	Sweet potatoes	0.0	0.0	20.5	8.0
Pulses, nuts and seeds		76.6	90.8	64.8	92.3
	Bambara nuts	16.0	28.8^a^	32.0	32.0^a^
	Dried beans	30.3	50.0^a^	25.0	50.0^a^
	Groundnuts	30.3	12.0^a^	7.8	10.3^a^
Vegetables		187.9	408.0	413.8	633.4
	Green and yellow vegetables	182.6	392.0^a^	409.0	576.8^a^
	Other vegetables	5.3	16.0^a^	4.8	56.6^a^
Fruits		8.0	16.0	4.0	8.0
	Baobab powder	8.0	16.0^a^	4.0	8.0^a^
Meat, poultry and insects		20.0	20.1	20.0	25.0
	Beef	20.0	20.1^a^	20.0	25.0^a^
Eggs	Egg	0.0	0.0	0.0	0.0
Fish and seafood		42.2	62.3	32.0	111.5
	Fish, dried	0.0	0.0	0.0	62.0^a^
	Sardines, dried	42.2	62.3^a^	32.0	49.5^a^
Milk and dairy products		30.3	45.0	0.0	0.0
	Cow milk	30.3	45.0	0.0	0.0
Fats and oils		7.0	2.2	7.6	2.7
	Vegetable oil	7.0	2.2	7.6	2.7
Sweets		23.3	14.2	21.0	0.0
	Sugar	23.3	14.2	21.0	0.0
Spices, condiments and beverages		3.2	1.1	2.5	0.7
	Ginger	0.0	0.0	0.0	0.0
	Table salt	2.3	0.9	1.9	0.7
	Tea, black	0.9	0.2	0.6	0.0
Total cost TZS (USD)		3200 (1.6)	5000 (2.5)	2300 (1.2)	3600 (1.8)

^a^ 90th percentile upper limit constraint was reached for the food group and subgroup patterns

### Nutrient profiles for optimal and observed diets

The recommended nutrient intake (RNI) values for pregnant and lactating women aged 19–50 years were established. Thereafter, nutrient profiles for observed and optimised food patterns for both pregnant and lactating mothers were generated (Table 3). Based on reference intake values, our results showed that the number of nutrients for which the RNIs were not achieved in the observed food intake pattern was 12 for pregnant women and nine for lactating mothers. On the contrary, the number of nutrients for which the nutritional requirements were not achieved in the optimised food intake patterns was six for pregnant women and three for lactating mothers. The most limiting nutrients were folate and zinc for pregnant women; and iron, vitamin E, pantothenic acid, and potassium for both pregnant and lactating mothers.

**Table 3:** Nutrient contents between observed and optimised daily food intake patterns

	Pregnant mothers 19–50 years (*n* = 75)		Lactating mothers 19–50 years (*n* = 75)	
Nutrients	Observed^b^	Optimised	Observed^b^	Optimised
Energy (Kcal/day)	2713.3	2156.9	2721.4	2374.2
Protein (g/day)	80.0	95.8	72.2	99.4
Calcium (mg/day)	887.5^a^	1457.9	787.1^a^	1760.4
Iron (mg/day)	21.3^a^	24.7^a^	18.6^a^	26.1^a^
Zinc (mg/day)	11.8^a^	12.3^a^	80.8	75.2
Copper (mg/day)	2.8	2.8	2.3	2.7
Magnesium (mg/day)	616.8	668.9	563.8	711.6
Manganese (mg/day)	12.8	13.3	9.5	11.6
Phosphorus (mg/day)	1758.8	2004.1	1461.2	1927.4
Potassium (mg/day)	2682.4^a^	3469.7^a^	2996.1^a^	5690.2
Sodium (g/day)	1009.1	284.7	1319.4	348.2
Folate (μg/day)	255.7^a^	442.2^a^	361.7^a^	752.9
Niacin (mgNE/day)	17.6^a^	18.9	62.6	21.5
Vitamin A (μgRE/day)	275.8^a^	1167.3	933.3	2552.0
Vitamin B1 (mg/day)	2.2	2.5	2.0	2.5
Vitamin B2 (mg/day)	3.2	7.8	4.7	9.7
Vitamin B6 (mg/day)	1.7^a^	2.0	1.9^a^	3.4
Vitamin B12 (μg/day)	2.7	6.2	2.1^a^	6.4
Vitamin C (mg/day)	43.6^a^	121.0	99.0	253.0
Vitamin D (μg/day)	0.6^a^	1.5^a^	2.1^a^	5.7
Vitamin E (mg/day)	4.0^a^	7.4^a^	6.5^a^	10.3^a^
Pantothenic acid (mg/day)	5.5^a^	5.3^a^	4.2^a^	5.0^a^

^a^Nutrients not meeting the recommended nutrient intakes (RNIs)

^b^Observed intake of nutrients in each group was based on the median population intake of nutrients

## Discussion

Individual-specific food intake patterns that met a set of 15 nutrient recommendations for pregnant women, and 19 nutrients RNIs for lactating mothers were designed using linear goal programming given the RNIs for pregnant and lactating women aged 19–50 years. The present analysis indicates that nutrient recommendations can be translated into realistic and adequate dietary intake patterns with a slight modification of existing feeding practices among pregnant and lactating women in rural settings. Our optimal food patterns ranged within 10th and 90th percentile limits of the observed habitual dietary intakes at food group level (Table 2). This implies that our optimal dietary patterns did not significantly deviate from cultural eating habits of the study population. On the other hand, intakes of some foods such as vegetables, fruits and animal products exceeded the upper limit (90th percentile). The reason for such exception was that consumption of these nutrient-dense foods was very low in both study groups. These findings are similar to those reported by Okubo and colleagues on optimal food intake patterns designed from local foods to achieve nutritional goals for Japanese adults [21].

In the present analysis, we further observed that dietary modifications to achieve the required nutritional goals for lactating mothers were greater than that of pregnant women in pulses, nuts and seeds; meat and poultry; and fish. On the other hand, dietary modifications to meet recommended nutrient intakes for lactating mother were observed to be lower than that of pregnant women in fruits and vegetables. An increase of 19% and 42% in pulses, nuts and seeds, for instance, was needed for pregnant women and lactating mothers, respectively. Likewise, foods of animal source such as meat and poultry needed to be increased by 0.5% for pregnant women and 25% for lactating mothers. Also, an increase of 48% and 248% in fish and seafood was needed for pregnant women and lactating mothers, respectively. On the other hand, an increase of 117% and 53% in vegetables was needed for pregnant women and lactating mothers, respectively. Similarly, fruits needed to be increased by 100% for pregnant women and 50% for lactating mothers. The observed differences are in part consistent with those reported in previous studies on dietary changes needed to achieve nutritional goals for each individual in Asian and French populations [21, 28].

The reasons for the observed differences in the degree of dietary modifications between pregnant women and lactating mothers are so far unclear. However, these differences might be explained based on the nutritional needs per physiological state of the woman. For example, the energy demand during lactation exceeds pregnancy demands by approximately 200 kcal/day [6]. Furthermore, the recommended nutrient intake (RNI) for most micronutrients which are based on amounts secreted in breast milk are higher for lactating mothers compared to pregnant women [6]. One notable exception is the RNIs for iron and folate, which are lower during lactation compared to pregnancy. In addition, the observed dietary adequacy for the limiting nutrients varied across the groups. For example, the achieved RNI adequacy for iron in the observed diets was lower (62%) in lactating mothers than in pregnant women (71%). On the other hand, the achieved RNI adequacy for folate (43%) in the observed diets was lower in pregnant women than in lactating mothers (72%). Thus, meeting daily intake recommendations for iron in lactating mothers needed a substantial increase in iron-rich foods such as pulses, nuts and seeds; foods of animal origin such as poultry and fish. Nevertheless, achieving daily intake for folate in pregnant mothers needed significant increase in foods like fruits and vegetables that are usually rich in dietary folate. However, all these modifications have a cost implication on food purchase for each individual. For example, both the pregnant women and lactating mothers would need to increase more than 50% of the observed cost on food purchases per day (Table 2), which may not be feasible especially, in poor families. Few studies have investigated differences in required dietary modifications based on amount of food intake, age and physiological states of women, a situation that hindered the comparison of our findings with those of other studies. Further studies are therefore, needed to confirm consistency of our findings in other populations.

On the contrary, a reduction of refined cereal grains was needed for the expense of achieving at least 10th percentile intake of whole cereal grains for both pregnant and lactating women. Furthermore, the developed optimised dietary patterns required a zero intake of other foods like sweets and some spices so as to achieve at least 10th percentile intake of oils and fats in both groups. These findings are generally consistent with food guidelines set by the Ministry of Health in Canada [29]. Standard dietary guidelines for pregnant and lactating women in Tanzania are not well documented, making it difficult to compare our modelled dietary patterns with those of other studies. The present study might therefore, contribute to the development of standard dietary guidelines for pregnant and lactating mothers in Tanzania.

Our present analysis found that only 45% and 59% of all studied nutrients met the RNI using the observed food patterns for pregnant and lactating mothers, respectively. On the other hand, the number of nutrients that achieved recommended intakes raised from 45% to 68% for pregnant women, and 59% to 86% for lactating mothers after using diet optimisation models. This implies that our modelled diets from local foods could address the challenge of undernutrition among the pregnant and lactating women aged 19–50 years in rural settings. However, our optimal food patterns contained exactly 100% RNIs of niacin and vitamin B6, indicating that these are also limiting nutrients in the studied population. In addition, iron, zinc, folate, vitamin E, and pantothenic acid requirements for pregnant women could not be met using optimal diets based on local foods. Poor iron status of the woman during conception may lead to postpartum anemia, and insufficient iron stores for the breast-feeding infant in the first six months of age [30]. Similarly, inadequate folate status during pregnancy may cause neural tube defects to infants. Some other risks associated with impaired status of folate during pregnancy include premature delivery, low birth weight and stillbirth [31]. The observed inadequacies of essential micronutrients underscores the need to set an effective nutrition intervention that can meet the RNIs for the identified nutrient gaps in the study area. Our findings are generally consistent with previous reports on the use of diet optimisation models to achieve nutritional goals of target groups [21, 28, 32, 33].

As in other previous studied populations such as Guatemala, French, American and Japanese populations, we identified iron, zinc, folate, pantothenic acid, vitamin D, and vitamin E as the most difficult constraints to achieve [21, 33]. Niacin, which was regarded as one of the limiting nutrients in our study, was rarely mentioned as a problem nutrient in previous studies of other countries. The differences in farming systems and feeding practices might be a source of the observed variability in limiting nutrients among the countries. In addition, dietary standards used can potentially cause the variation in problem nutrients among the countries. For example, the recommended intake of niacin in Japan is 13-15 mg/day for pregnant and lactating women [34], compared to 17-18 mg/day in the USA and Canada [35, 36] and 11 mg/day in France [28]. These results show that optimal dietary intake patterns should be dependent on dietary standard values selected as nutritional constraints for a given population.

Generally, our LP analysis showed that locally available foods can be optimised to improve RNIs for key problem nutrients in developing countries. The final optimal food patterns contained a variety of ingredients such as wholegrain cereals (maize flour, millet, sorghum, rice); roots and tubers (Irish potatoes, sweet potatoes); fish (dried fish and sardines); indigenous fruits (baobab powder); pulses, nuts and seeds; green and yellow vegetables; and meat that can be sourced locally. Also, the modelled formula contained a mixture of ingredients that can enhance bioavailability of iron, zinc and vitamin A. The baobab powder in the mixture, for example, is a rich source of ascorbic acid- a well-known iron absorption enhancer [37, 38]. Fish and poultry meat can improve the bioavailability of iron and zinc in the modelled formula [37]. Also, seeds and nuts in the optimal formula can provide essential oils that can enhance the bioavailability of beta-carotene in the modelled formulation [39]. On the other hand, the optimal formula may contain iron and zinc absorption inhibitors such as phytates in wholegrain cereals, seeds and legumes. Experimental studies are therefore needed to establish the actual biological value of essential micronutrients in the modelled formula. Any future changes in price and availability of local ingredients can be modified and accommodated using linear goal programming techniques used in the current study. The goal is to ensure that our modeled diets are available in the affected low income communities. The present analysis creates a baseline for developing food composition tables for locally available foods and establish specific standard dietary guidelines for pregnant and lactating women in the study area.

Even though the study had several strengths, it warrants some weaknesses. The nutritional information for some local foods were not available in the current Tanzania food composition tables. So, we had to use food composition databases from other countries. Nutrient composition in foods is known to vary with the variety and location, this might have affected our analysis, especially, if the nutrient database of candidate ingredients in the final formulations was not accurate [40, 41]. Likewise, we used the median portion sizes above the 90th percentile for some ingredients under the assumption that these foods could be successfully promoted in the community. But the practicability of adopting the modelled food patterns may be a challenge at population level. Another limitation is that our sample size was relatively small for both pregnant women (*n* = 75) and lactating mothers (*n* = 75). Therefore, our estimates on the distribution of food consumption levels might not be stable. Additionally, our analysis did not take seasonality into account. Thus, dietary patterns identified refer basically to the dry season of the study area. Comprehensive studies, therefore, are needed to verify the effect of seasonality on food availability and feeding practices in the study population.

In conclusion, our optimal dietary patterns designed from locally available foods improved the nutritional goals for pregnant and lactating mothers**.** The modelled diets met the RNIs for 19 selected nutrients in pregnant women and 15 selected nutrients for lactating mothers and achieved more than 80% RNI for iron, which was identified as one of the problem nutrients in both pregnant and lactating mothers in the studied population. This means that if nutrition practitioners are informed about the available food choices in each locality, it is possible for them to devise a feasible nutrition solution at household level and scale it up at community level. Based on the present study, an increase of about 50% of the observed food budget per mother is needed to improve dietary recommendations for problem nutrients among pregnant and lactating women aged 19–50 years in rural households. The findings from this study underscore the need for additional cost-effective interventions to ensure adequate intake of nutrients like iron, folate, and vitamin E that could not meet 100% RNI even after diet optimisation. One possible intervention could be the enhancement of local availability of affordable nutrient-dense foods for pregnant and lactating mothers in low income settings.

## Abbreviations

BMI: Body mass index
LP: Linear programming
RNI: Recommended nutrient intake
SSA: Sub-Saharan Africa
UL: Upper limit
WDR: Weighed dietary record
